# A central limit theorem for integer partitions into small powers

**DOI:** 10.1007/s00605-023-01926-y

**Published:** 2023-12-15

**Authors:** Gabriel F. Lipnik, Manfred G. Madritsch, Robert F. Tichy

**Affiliations:** 1 Institute of Analysis and Number Theory, Graz University of Technology, 8010 Graz, Austria; 2 Université de Lorraine, IECL, CNRS, F-54000 Nancy, France

**Keywords:** Integer partitions, Partition function, Central limit theorem, Saddle-point method, Mellin transform, 11P82, 05A17, 60F05

## Abstract

The study of the well-known partition function *p*(*n*) counting the number of solutions to $n = a_{1} + \dots + a_{\ell }$ with integers $1 \le a_{1} \le \dots \le a_{\ell }$ has a long history in number theory and combinatorics. In this paper, we study a variant, namely partitions of integers into

$$\begin{aligned} n=\left\lfloor a_1^\alpha \right\rfloor +\cdots +\left\lfloor a_\ell ^\alpha \right\rfloor \end{aligned}$$

with $1\le a_1< \cdots < a_\ell$ and some fixed $0< \alpha < 1$. In particular, we prove a central limit theorem for the number of summands in such partitions, using the saddle-point method.

## Introduction

For a positive integer *n*, let *f*(*n*) denote the number of unordered factorizations as products of integer factors greater than 1. Balasubramanian and Luca [1] considered the set

$$\begin{aligned} \mathcal {F}(x)=\{{m}|{m\le x,\, m=f(n)\text { for some }n}.\}\end{aligned}$$

In order to provide an upper bound for ${\left|\mathcal {F}(x) \right|}$, they had to analyse the number *q*(*n*) of partitions of *n* of the form

$$\begin{aligned} n=\left\lfloor \sqrt{a_1}\right\rfloor +\cdots +\left\lfloor \sqrt{a_\ell }\right\rfloor \end{aligned}$$

with integers $1\le a_1\le \cdots \le a_\ell$, where $\left\lfloor x\right\rfloor$ denotes the integer part of *x*.

Chen and Li [2] proved a similar result, and Luca and Ralaivaosaona [19] refined the previous results to obtain the asymptotic formula

$$\begin{aligned} q(n)\sim Kn^{-8/9}\exp \biggl (\frac{6\zeta (3)^{1/3}}{4^{2/3}}n^{2/3}+\frac{\zeta (2)}{(4\zeta (3))^{1/3}}n^{1/3}\biggr ),\end{aligned}$$

where

$$\begin{aligned}K=\frac{(4\zeta (3))^{7/18}}{\pi A^2\sqrt{12}}\exp \biggl (\frac{4\zeta (3)-\zeta (2)^2}{24\zeta (3)}\biggr )\end{aligned}$$

and *A* is the Glaisher–Kinkelin constant. Li and Chen [16, 17] extended the result to arbitrary powers $0<\alpha <1$ not being of the form $\alpha =1/m$ for a positive integer *m*. Finally, Li and Wu [18] considered the case of $\alpha =1/m$. They obtained a complete expansion along lines similar to the one in Tenenbaum, Wu and Li [25] as well as in Debruyne and Tenenbaum [6].

In the present paper, we take a different point of view. For fixed $0<\alpha <1$, we consider the distribution of the length of restricted $\alpha$-partitions. A restricted $\alpha$-partition of *n* is a representation of *n* of the form

$$\begin{aligned} n=\left\lfloor a_1^\alpha \right\rfloor +\cdots +\left\lfloor a_\ell ^\alpha \right\rfloor \end{aligned}$$

with integers $1\le a_1< a_2< \cdots < a_\ell$, and $\ell$ is called its length. We denote by $q(n,\ell )$ the number of restricted partitions of length $\ell$.

In the literature also unrestricted partitions are considered. We call a partition unrestricted if the $a_i$’s can be equal, *i.e.* $1\le a_1\le a_2\le \cdots \le a_\ell$. Analogously we denote by *p*(*n*) and $p(n,\ell )$ the number of unrestricted partitions as well as the number of unrestricted partitions of length $\ell$.

The asymptotic analysis of partition problems has its origin in the work of Hardy and Ramanujan, see for instance [13]. Their proof is based on properties of elliptic modular functions and later Rademacher [22] and followers could achieve full asymptotic expansions by this method. Ingham [15] developed a more elementary approach (comparable to our method) for the asymptotic analysis of certain partition problems. Here we also want to mention the work Chern [3], who obtained the asymptotics with explicit constants for the partition function in the case $\alpha =\tfrac{1}{2}$.

Erdős and Lehner [7] were among the first to consider the distribution of the length of a partition. In particular they considered the ratio $p(n,\ell )/p(n)$, where $\ell =(2\pi ^2/3)^{-\frac{1}{2}}\sqrt{n}\log n+x\sqrt{n}$ is a function of *n*. The study of distinct parts was introduced by Wilf [26]. Goh and Schmutz [11] proved a central limit theorem for the distribution of part sizes. Their result was extended by Schmutz [24] to multivariate cases under the Meinardus’ scheme (cf. Meinardus [21]). Hwang [14] provided an extended version with weaker necessary conditions to obtain limit theorems for the number of summands in a random partition (restricted and unrestricted).

While Meinardus’ original scheme can handle Dirichlet generating functions with a single pole on the positive real axis, Granovsky and Stark [12] and Chern [3] adapted the method for multiple poles on the real axis. Madritsch and Wagner [20] considered sets with digital restrictions, leading to a Dirichlet generating function having equidistant poles along a vertical line in the complex plane, and proved a central limit theorem. Motivated by a question in Hwang’s paper [14] Ralaivaosaona [23] established a central limit theorem for partitions in primes. In the present paper, we use a similar method for the case of multiple poles on the real line.

## Main Result

Let $0<\alpha <1$ be a fixed real number. We let $\Pi (n)$ denote the set of partitions of a positive integer *n* into parts $\left\lfloor a_j^{\alpha }\right\rfloor$ where each $a_j$ occurs at most once. These partitions are called (restricted) $\alpha$-partitions for short. Furthermore, let $q(n)={\left|\Pi (n) \right|}$ be the cardinality of the set $\Pi (n)$. Finally, we let $\Pi (n,k)$ denote the subset of partitions $\Pi (n)$ whose length (number of summands) is *k* and $q(n,k)={\left|\Pi (n,k) \right|}$ is its cardinality.

In the present work, we consider the random variable $\varpi _n$ counting the number of summands in a random $\alpha$-partition of *n*. The probability distribution of $\varpi _n$ is given by ${\mathbb {P}}(\varpi _n = k)= q(n,k)/q(n)$. In order to obtain a central limit theorem for $\varpi _n$, we have to carefully analyse the associated bivariate generating function *Q*(*z*, *u*), which is given by

$$\begin{aligned} Q(z,u)=1+\sum _{n\ge 1}\sum _{k\ge 1} q(n,k)u^kz^n.\end{aligned}$$

Furthermore, for a fixed integer $k \ge 1$, we let *g*(*k*) denote the number of integers $n \ge 1$ satisfying $\left\lfloor n^\alpha \right\rfloor =k$, i.e.,with $\beta :=1/\alpha$. Then the following lemma holds.

### Lemma 1

With the notation above, we have

$$\begin{aligned} Q(z, u) =\prod _{k\ge 1}\bigl (1+uz^k\bigr )^{g(k)} =1+\sum _{n\ge 1}q(n){\mathbb {E}}(u^{\varpi _n})z^n. \end{aligned}$$

### Proof

By the definition of *g*(*k*), we have $\left\lfloor a_{j}^\alpha \right\rfloor =k$ for exactly *g*(*k*) different integers $a_{j}$. Thus, it follows that

$$\begin{aligned}Q(z,u)=\prod _{k\ge 1}\bigl (1+uz^k\bigr )^{g(k)}.\end{aligned}$$

Furthermore, it holds that

$$\begin{aligned} Q(z,u)&=1+\sum _{n\ge 1}\sum _{k\ge 1}q(n,k)u^kz^n\\&=1+\sum _{n\ge 1}q(n)\sum _{k\ge 1}\frac{q(n,k)u^k}{q(n)} z^n\\&=1+\sum _{n\ge 1}q(n){\mathbb {E}}(u^{\varpi _n}) z^n. \end{aligned}$$

$$\square $$

Now we can state the main theorem of this work as follows.

### Theorem 2

Let $0<\alpha <1$ and let $\varpi _n$ be the random variable counting the number of summands in a random restricted partition of *n* into $\alpha$-powers. Then $\varpi _n$ is asymptotically normally distributed with mean ${\mathbb {E}}(\varpi _n)\sim \mu _n$ and variance ${\mathbb {V}}(\varpi _n)\sim \sigma _n^2$, i.e.,

$$\begin{aligned} {\mathbb {P}}\left( \frac{\varpi _{n} - \mu _n}{\sigma _n}< x\right) =\frac{1}{\sqrt{2\pi }}\int _{-\infty }^x e^{-t^2/2}\textrm{d}t+o(1), \end{aligned}$$

uniformly for all *x* as $n\rightarrow \infty$. The mean $\mu _n$ and the variance $\sigma _n^2$ are given by

2.1 $$\begin{aligned} \mu _n = \sum _{k\ge 1}\frac{g(k)}{e^{\eta k}+1} \sim c_{1}n^{1/(\alpha + 1)} \end{aligned}$$

and

2.2 $$\begin{aligned} \sigma _n^2 = \sum _{k\ge 1}\frac{g(k)e^{\eta k}}{(e^{\eta k}+1)^2} -\frac{\left( \sum _{k\ge 1}\frac{g(k)ke^{\eta k}}{(e^{\eta k}+1)^2}\right) ^2}{\sum _{k\ge 1}\frac{g(k)k^2e^{\eta k}}{(e^{\eta k}+1)^2}} \sim c_{2}n^{1/(\alpha + 1)}, \end{aligned}$$

where $\eta$ is the implicit solution of

$$\begin{aligned} n=\sum _{k\ge 1}\frac{k}{e^{\eta k}+1}. \end{aligned}$$

Explicit formulæ for the occurring constants $c_{1}$ and $c_{2}$ are given in (4.12) and (4.13), respectively.

Finally, the tails of the distribution satisfy the exponential bounds

$$\begin{aligned} {\mathbb {P}}\Bigl (\frac{\varpi _{n} - \mu _n}{\sigma _n}\!\ge \! x\Bigr ) \le {\left\{ \begin{array}{ll} e^{-x^2/2}\left( 1+\mathcal {O}((\log n)^{-3})\right) &{}\text {if}\,\, 0\le x\le n^{1/(6\alpha +6)}/\log n,\\ e^{-n^{1/(6\alpha +6)}x/2}\left( 1+\mathcal {O}((\log n)^{-3})\right) &{}\text {if}\,\, x\ge n^{1/(6\alpha +6)}/\log n, \end{array}\right. } \end{aligned}$$

and the analogous inequalities also hold for ${\mathbb {P}}\bigl (\frac{\varpi _n - \mu _n}{\sigma _n}\le -x\bigr )$.

This result fits into the series of other results on partitions in integers of the form $\lfloor k^\alpha \rfloor$ with $k\ge 1$. In particular, if $\alpha =1$, then we have the classic case of partitions and Erdős and Lehner [7] showed that $\mu _n\sim cn^{1/2}$. For $\alpha >1$, not every integer has a representation of the form $\lfloor k^\alpha \rfloor$ and there are gaps in the set $\{\left\lfloor k^{\alpha }\right\rfloor |{k\in {\mathbb {N}}}\}$. This led Hwang [14] to the result $\mu _n\sim cn^{1/(\alpha +1)}$. Consequently, our result $\mu _n\sim c_1n^{1/(\alpha +1)}$ seems to be a natural extension of these results.

One of the main difficulties of the case $0<\alpha <1$ lies in the special structure of the function *g*(*k*). In particular, if $\alpha =1/m$ with $m\ge 2$ being an integer, then the parts of the partitions are *m*th roots and *g*(*k*) is given by the polynomial

$$\begin{aligned}g(k)=(k+1)^m - k^m=\sum _{r=0}^{m-1}\left( {\begin{array}{c}m\\ r\end{array}}\right) k^r.\end{aligned}$$

However, in the general case of $\alpha \not \in {\mathbb {Q}}$, we have an additional error term (see (3.8)) of which no explicit form is known. This makes the analysis more involved.

Finally, we want to mention that a local version of this central limit theorem is the topic of a subsequent project. In particular, it seems that the above mentioned error term of the function *g*(*k*) needs further considerations in this case.

## Main Idea, Outline and Tools for the Proof

The proof of our main theorem consists of analytic and probabilistic parts. In the analytic part, we use Mellin transforms and the saddle-point method. The probabilistic part is based on the use of Curtiss’ theorem for moment-generating functions. Before we give the details of the proof, this section is dedicated to give an overview of the main techniques and tools.

We first note that the central limit theorem for the random variable $\varpi _n$ is equivalent to the fact that the normalized moment-generating function $M_n(t)={\mathbb {E}}(e^{(\varpi _n-\mu _n)t/\sigma _n})$ tends to $e^{t^2/2}$ for $t\rightarrow \infty$. Consequently, we will show this limit. Furthermore, the mentioned tail estimates can be obtained by the Chernoff bound.

Since $\mu _n/\sigma _n$ is constant, we have ${\mathbb {E}}(e^{(\varpi _n-\mu _n)t/\sigma _n})=e^{-\mu _n/\sigma _n}{\mathbb {E}}(e^{\varpi _n t/\sigma _n})$. We recall that by Lemma 1, the probability-generating function ${\mathbb {E}}(u^{\varpi _n})$ is given by

$$\begin{aligned}{\mathbb {E}}(u^{\varpi _n})=\frac{[z^n]Q(z,u)}{[z^n]Q(z,1)}.\end{aligned}$$

In other words, it is sufficient for our purpose to obtain the coefficient of $z^n$ in *Q*(*z*, *u*). By Cauchy’s integral formula, we derive

$$\begin{aligned} Q_n(u):=[z^n]Q(z,u) =\frac{1}{2\pi i}\oint _{\left| z\right| =e^{-r}} z^{-n-1}Q(z,u)\textrm{d}{z}. \end{aligned}$$

A standard transformation yields for $r > 0$ that

3.1 $$\begin{aligned} Q_n(u) = \frac{e^{nr}}{2\pi }\int _{-\pi }^{\pi }\exp \bigl (int+f(r+it,u)\bigr )\textrm{d}{t} \end{aligned}$$

with

$$\begin{aligned} f(\tau ,u):=\log Q\left( e^{-\tau },u\right) =\sum _{k\ge 1} g(k)\log \bigl (1+ue^{-k\tau }\bigr ). \end{aligned}$$

For the integral in (3.1), we use the well-known saddle-point method, also known as the method of steepest decent. The main application of this method is to obtain estimates for integrals of the form

$$\begin{aligned}\int _{-\pi }^\pi e^{g(r+it)}\textrm{d}t\end{aligned}$$

for some suitable function *g*. We choose $t_n>0$ in order to split up the integral into two parts, one near the positive real axis and the other one for the rest, i.e.,

3.2 $$\begin{aligned} \int _{-\pi }^\pi e^{g(r+it)}\textrm{d}{t} =\int _{\left| t\right| \le t_n} e^{g(r+it)}\textrm{d}{t} +\int _{t_n<\left| t\right| \le \pi } e^{g(r+it)}\textrm{d}{t}. \end{aligned}$$

For the second integral, we compare the contribution of the integrand with the contribution from the real line, i.e., we estimate $e^{g(r+it)-\Re (g(r))}$. This will contribute to the error term.

For the first integral in (3.2), we use a third order Taylor expansion of $g(r+it)$ around $t=0$, which is

3.3 $$\begin{aligned} g(r+it)=g(r) + it g'(r)-\frac{t^2}{2}g''(r)+\mathcal {O}\Bigl (\sup _t\left| t^3g'''(r+it)\right| \Bigr ). \end{aligned}$$

Now we choose *r* such that the first derivative $g'(r)$ vanishes. Then the remaining integral is given by

$$\begin{aligned}\int _{\left| t\right|<t_n} e^{g(r+it)}\textrm{d}{t} =e^{g(r)}\int _{\left| t\right| <t_n} e^{-\frac{t^2}{2}g''(r)}\Bigl (1+\mathcal {O}\Bigl (\sup _t \left| t^3g'''(r+it)\right| \Bigr )\Bigr )\textrm{d}{t}.\end{aligned}$$

Now the integrand is that of a Gaussian integral and so we add the missing part. The Gaussian integral contributes to the main part, and we need to analyse $g''(r)$ and $g'''(r)$ in order to show that all our transformations and estimates are valid. For more details on the saddle-point method, we refer the interested reader to Flajolet and Sedgewick [10, Chapter VIII].

The estimates for $g''(r)$ and $g'''(r)$ are based on singular analysis using the well-known Mellin transform. The Mellin transform $h^{*}(s)$ of a function *h* is defined by

$$\begin{aligned} h^*(s)={{\,\mathrm{\mathcal {M}}\,}}[h;s]=\int _0^\infty h(t)t^{s-1}\textrm{d}{t}.\end{aligned}$$

The most important property for our considerations is the so called rescaling rule, which is given by

$$\begin{aligned}{{\,\mathrm{\mathcal {M}}\,}}\biggl [\sum _{k}\lambda _kh(\mu _kx);s\biggr ]=\biggl (\sum _{k}\frac{\lambda _k}{\mu _k^s}\biggr )h^*(s);\end{aligned}$$

see [8, Theorem 1]. This provides a link between a generating function and its Dirichlet generating function. For a detailed account on this integral transform, we refer the interested reader to the work of Flajolet, Gourdon and Dumas [8] and to the work of Flajolet, Grabner, Kirschenhofer, Prodinger and Tichy [9].

Let $\delta >0$. Throughout the rest of our paper we assume $\delta \le u\le \delta ^{-1}$ and by “uniformly in *u*” we always mean “uniformly as $\delta \le u\le \delta ^{-1}$”. Then in our case we have for the Mellin transform of $f(\tau ,u)$ with respect to $\tau$ that

$$\begin{aligned}{{\,\mathrm{\mathcal {M}}\,}}\left[ \sum _{k\ge 1} g(k)\log \left( 1+ue^{-k\tau }\right) ;s\right] =D(s)Y(s,u),\end{aligned}$$

where

3.4 $$\begin{aligned} D(s)=\sum _{k\ge 1} \frac{g(k)}{k^s} \end{aligned}$$

is the associated Dirichlet series and

3.5 $$\begin{aligned} Y(s)=\int _0^\infty \left( 1+ue^{-\tau }\right) \tau ^{s-1}\textrm{d}\tau \end{aligned}$$

is the Mellin transform of $\tau \mapsto \left( 1+ue^{-\tau }\right)$.

The central advantage of the Mellin transform is not necessarily the transformation itself but moreover the converse mapping, where we consider the singularities of the transformed function providing the asymptotic expansion.

### Theorem 3

[Converse Mapping [8, Theorem 4]] Let *f*(*x*) be continuous in $(0,+\infty )$ with Mellin transform $f^*(s)$ having a nonempty fundamental strip $\langle \alpha ,\beta \rangle$. Assume that $f^*(s)$ admits a meromorphic continuation to the strip $\langle \gamma , \beta \rangle$ for some $\gamma <\alpha$ with a finite number of poles there, and is analytic on $\Re (s)=\gamma$. Assume also that there exists a real number $\eta \in (\alpha ,\beta )$ such that

3.6 $$\begin{aligned} f^*(s)=\mathcal {O}({\left|s \right|}^{-r}) \end{aligned}$$

with $r > 1$ as ${\left|s \right|}\rightarrow \infty$ in $\gamma \le \Re (s)\le \eta$. If $f^*(s)$ admits the singular expansion

3.7 $$\begin{aligned} f^*(s)\asymp \sum _{(\xi ,k)\in A}d_{\xi ,k}\frac{1}{(s-\xi )^k} \end{aligned}$$

for $s\in \langle \gamma ,\alpha \rangle$, then an asymptotic expansion of *f*(*x*) at 0 is given by

$$\begin{aligned}f(x)=\sum _{(\xi ,k)\in A}d_{\xi ,k}\left( \frac{(-1)^{k-1}}{(k-1)!}x^{-\xi }(\log x)^{k-1}\right) +\mathcal {O}(x^{-\gamma }).\end{aligned}$$

Thus in our case we have to consider the singularities of the associated Dirichlet series *D*(*s*) and the function *Y*(*s*, *u*). On the one hand we note that3.8where *m* is the integer such that $m<\beta \le m+1$. Plugging this into (3.4) yields

$$\begin{aligned} D(s)=\sum _{\nu =1}^{m}\left( {\begin{array}{c}\beta \\ \nu \end{array}}\right) \zeta (s-\beta +\nu )+\tilde{D}(s), \end{aligned}$$

where $\tilde{D}(s)$ has no pole with $\Re (s)>1$ and

$$\begin{aligned} \zeta (s)=\sum _{k\ge 1}k^{-s} \end{aligned}$$

is the Riemann zeta function. On the other hand

$$\begin{aligned} Y(s,u) =\int _0^\infty \left( 1+ue^{-\tau }\right) \tau ^{s-1}\textrm{d}\tau =\int _0^\infty \sum _{\ell \ge 1}\frac{(-u)^\ell }{\ell }e^{-\ell \tau }\tau ^{s-1} \textrm{d}\tau ={{\,\textrm{Li}\,}}_{s+1}(-u) \Gamma (s), \end{aligned}$$

where

$$\begin{aligned} {{\,\textrm{Li}\,}}_s(z)=\sum _{n\ge 1}\frac{z^n}{n^s} \text { and } \Gamma (s)=\int _0^\infty e^{-x}x^{s-1}\textrm{d}{x} \end{aligned}$$

are the polylogarithm and the Gamma function, respectively.

Now in order to apply converse mapping, we need to show that (3.6) as well as (3.7) are both fulfilled for these three functions. Stirling’s formula yields for the Gamma function that

$$\begin{aligned}{\left|\Gamma (x+iy) \right|}=\sqrt{2\pi }{\left|y \right|}^{x-1/2}e^{-\pi {\left|y \right|}/2}\left( 1+\mathcal {O}_{a,b}(1/y)\right) \end{aligned}$$

for $a\le x\le b$ and ${\left|y \right|}\ge 1$. Furthermore, the Riemann zeta function satisfies

$$\begin{aligned}\zeta (x+iy)\ll _{a,b}1+{\left|y \right|}^A\end{aligned}$$

for suitable $A=A(a,b)$, $a\le x\le b$ and ${\left|y \right|}\ge 1$. For the polylogarithm we follow the ideas of Flajolet and Sedgewick [10, VI.8]. This is a good application of the converse mapping, so we want to reproduce it here: First of all, let $w=-\log z$ and define the function

$$\begin{aligned}\Lambda (w):={{\,\textrm{Li}\,}}_{\alpha }(e^{-w})=\sum _{n\ge 1}\frac{e^{-nw}}{n^\alpha }.\end{aligned}$$

This is a harmonic sum and so we apply Mellin transform theory. The Mellin transform of $\Lambda (w)$ satisfies

$$\begin{aligned} \Lambda ^*(s) =\int _{0}^\infty \sum _{n\ge 1}\frac{e^{-nw}}{n^\alpha }w^{s-1}\textrm{d}w =\sum _{n\ge 1}\frac{1}{n^{\alpha +s}} \int _{0}^\infty e^{-v}v^{s-1}\textrm{d}v =\zeta (s+\alpha )\Gamma (s) \end{aligned}$$

for $\Re (s)>\max (0,1-\alpha )$. The Gamma function has simple poles at the negative integers and $\zeta (s+\alpha )$ has a simple pole at $1-\alpha$. Thus, the application of converse mapping (Theorem 3) yields

$$\begin{aligned} {{\,\textrm{Li}\,}}_\alpha (z)=\Gamma (1-\alpha )w^{\alpha -1} +\sum _{j\ge 0}\frac{(-1)^j}{j!}\zeta (\alpha -j)w^j \end{aligned}$$

with

$$\begin{aligned} w=\sum _{\ell \ge 1}\frac{(1-z)^\ell }{\ell }. \end{aligned}$$

Using these estimates in the converse mapping, we obtain an asymptotic formula for $Q_n(u)$ of the form

$$\begin{aligned} Q_n(u)=\frac{e^{nr+f(u,r)}}{\sqrt{2\pi B}}\bigl (1+\mathcal {O}\bigl (r^{2\beta /7}\bigr )\bigr ). \end{aligned}$$

Recall that

$$\begin{aligned}M_n(t)={\mathbb {E}}(e^{(\varpi _n-\mu _n)t/\sigma _n}) =\exp \Bigl (-\frac{\mu _nt}{\sigma _n}\Bigr ) {\mathbb {E}}(e^{t\varpi _n/\sigma _n}) =\exp \Bigl (-\frac{\mu _nt}{\sigma _n}\Bigr )\frac{Q_n(e^{t/\sigma _n})}{Q_n(1)}.\end{aligned}$$

Using implicit differentiation, we obtain a Taylor expansion for the moment-generating function, which yields

$$\begin{aligned} M_n(t) =\exp \left( \frac{t^2}{2}+\mathcal {O}\left( n^{2\beta /7}\right) \right) , \end{aligned}$$

proving the central limit theorem for $\varpi _n$. Finally, we will use the Chernoff bound for the tail estimates.

## Proof of the Main Result

To prove Theorem 2, we apply the method we have outlined in the previous section. As indicated above, we choose $r=r(n,u)$ such that the first derivative in (3.3) vanishes, i.e.,

$$\begin{aligned}\left. \frac{\partial \left( int+f(r+it,u)\right) }{\partial t}\right| _{t=0} =in-i\sum _{k\ge 1}\frac{kg(k)}{u^{-1}e^{kr}+1}=0. \end{aligned}$$

Since the sum is decreasing in *r*, we see that this equation has a unique solution, which is the saddle point. The main value of the integral in (3.1) lies around the positive real axis. We set $t_n=r^{1+3\beta /7}$ and split the integral into two ranges, namely into

$$\begin{aligned} Q_n(u) = I_1+I_2 \end{aligned}$$

where

$$\begin{aligned} I_1=\frac{e^{nr}}{2\pi }\int _{{\left|t \right|}\le t_n}\exp \bigl (int+f(r+it,u)\bigr )\textrm{d}{t} \end{aligned}$$

and

$$\begin{aligned} I_2=\frac{e^{nr}}{2\pi }\int _{t_n<{\left|t \right|}\le \pi }\exp \bigl (int+f(r+it,u)\bigr )\textrm{d}{t}. \end{aligned}$$

### Estimate of $I_1$

We start our considerations with the central integral $I_1$ and show the following lemma on its asymptotic behavior.

#### Lemma 4

Let $B^{2} = f_{\tau \tau }(r,u)$. Then we have

$$\begin{aligned} I_{1} = \frac{e^{nr+f(r, u)}}{2\pi }\left( \int _{-t_{n}}^{t_{n}} \exp \left( -\frac{B^2}{2}t^2\right) \textrm{d}{t}\right) \Bigl (1+\mathcal {O}\bigl (r^{2\beta /7}\bigr )\Bigr ) \end{aligned}$$

with

$$\begin{aligned} \int _{-t_{n}}^{t_{n}} \exp \left( -\frac{B^2}{2}t^2\right) \textrm{d}{t} = \frac{\sqrt{2\pi }}{B}+\mathcal {O}\biggl ( r^{-1-3\beta /7}B^{-2}\exp \biggl (-\frac{r^{-\beta /7}}{2}\biggr )\biggr ) \end{aligned}$$

uniformly in *u*.

Recall that by “uniformly in *u*” we always mean “uniformly as $\delta \le u\le \delta ^{-1}$”.

#### Proof

It holds that

4.1 $$\begin{aligned} f(r+it,u)=f(r,u)+f_\tau (r,u)it-\frac{f_{\tau \tau }(r,u)}{2}t^2+\mathcal {O}\Bigl (t^3\sup _{0\le t_0\le t}{\left|f_{\tau \tau \tau }(r+it_0,u) \right|}\Bigr ).\nonumber \\ \end{aligned}$$

Then from (3.8) we derive

4.2 $$\begin{aligned} \begin{aligned} f(\tau ,u)&=\sum _{k\ge 1}g(k)\log {(1+ue^{-k\tau })}\\&=-\sum _{k\ge 1}g(k)\sum _{\ell \ge 1}\frac{(-u)^\ell }{\ell }e^{-k\ell \tau }\\&=-\sum _{k\ge 1}\sum _{\nu =1}^m\left( {\begin{array}{c}\beta \\ \nu \end{array}}\right) k^{\beta -\nu }\sum _{\ell \ge 1}\frac{(-u)^\ell }{\ell }e^{-k\ell \tau } +\mathcal {O}\Biggl (\sum _{k\ge 1}\sum _{\ell \ge 1}\frac{(-u)^\ell }{\ell }e^{-k\ell \tau }\Biggr )\\&=-\sum _{\nu =1}^m\left( {\begin{array}{c}\beta \\ \nu \end{array}}\right) \sum _{k\ge 1}k^{\beta -\nu }\sum _{\ell \ge 1}\frac{(-u)^\ell }{\ell }e^{-k\ell \tau } +\mathcal {O}\Biggl (\sum _{k\ge 1}\sum _{\ell \ge 1}\frac{(-u)^\ell }{\ell }e^{-k\ell \tau }\Biggr ). \end{aligned}\nonumber \\ \end{aligned}$$

Note that in (4.2) the involved quantities may be complex numbers and the $\mathcal {O}$-notation $A=B+\mathcal {O}(C)$ in this case means that there is a constant $\gamma >0$ (only depending on $\beta =1/\alpha$) such that ${\left|A-B \right|}\le \gamma {\left|C \right|}$.

For $j\ge 0$, we analogously obtain

4.3 $$\begin{aligned} \frac{\partial ^j f}{\partial \tau ^j}(\tau , u)= & {} (-1)^{j+1}\sum _{\nu =1}^m\left( {\begin{array}{c}\beta \\ \nu \end{array}}\right) \sum _{k\ge 1}k^{\beta -\nu +j}\sum _{\ell \ge 1}(-u)^\ell \ell ^{j-1}e^{-k\ell \tau }\nonumber \\{} & {} +\mathcal {O}\Biggl (\sum _{k\ge 1}k^j\sum _{\ell \ge 1}(-u)^\ell \ell ^{j-1}e^{-k\ell \tau }\Biggr ). \end{aligned}$$

All infinite sums in (4.3) are of the form

$$\begin{aligned} h_{\gamma ,j}(\tau ,u)=\sum _{k\ge 1}k^{\gamma +j}\sum _{\ell \ge 1}(-u)^\ell \ell ^{j-1}e^{-k\ell \tau } \end{aligned}$$

with $\gamma > 0$. Let $H_{\gamma ,j}(s,u)$ denote the Mellin transform of $h_{\gamma ,j}(\tau ,u)$ with respect to $\tau$, then $H_{\gamma ,j}(s,u)$ is given by

$$\begin{aligned} H_{\gamma ,j}(s,u)&=\zeta (s-\gamma -j){{\,\textrm{Li}\,}}_{s-j+1}(-u)\Gamma (s). \end{aligned}$$

The function $H_{\gamma ,j}(s,u)$ converges for $\Re s>\gamma +j+2$ and its only pole in the range $j+\frac{1}{2}\le \Re s\le \gamma +j+2$ is that of $\zeta (s-\gamma -j)$ at $s=\gamma +j+1$. The Riemann zeta function and the polylogarithm grow only polynomially, whereas the Gamma function decreases exponentially on every vertical line in the complex plane; see Section 3. Thus, we can apply converse mapping (Theorem 3) and obtain

$$\begin{aligned}h_{\gamma ,j}(\tau ,u)={{\,\textrm{Li}\,}}_{\gamma +2}(-u)\Gamma (\gamma +j+1)\tau ^{-\gamma -j-1}+\mathcal {O}\bigl (\tau ^{-j-\frac{1}{2}}\bigr ).\end{aligned}$$

By plugging everything into (4.3), we obtain

4.4 $$\begin{aligned} \begin{aligned}&\frac{\partial ^jf}{\partial \tau ^j}(\tau ,u) =(-1)^{j+1}\sum _{\nu =1}^m\left( {\begin{array}{c}\beta \\ \nu \end{array}}\right) h_{\beta -\nu ,j}(\tau ,u) +\mathcal {O}\left( h_{0,j}(\tau ,u)\right) \\&\quad =(-1)^{j+1}\sum _{\nu =1}^m\left( {\begin{array}{c}\beta \\ \nu \end{array}}\right) {{\,\textrm{Li}\,}}_{\beta -\nu +2}(-u)\Gamma (\beta -\nu +j+1)\tau ^{-\beta +\nu -j-1} +\mathcal {O}\left( \tau ^{-j-1}\right) . \end{aligned}\nonumber \\ \end{aligned}$$

For the saddle point *n*, this results in

4.5 $$\begin{aligned} n = -f_{\tau }(r, u) = -\sum _{\nu = 1}^{m}\left( {\begin{array}{c}\beta \\ \nu \end{array}}\right) {{\,\textrm{Li}\,}}_{\beta -\nu +2}(-u)\Gamma (\beta -\nu +2) r^{-\beta +\nu -2} + \mathcal {O}\bigl (r^{-2}\bigr ),\nonumber \\ \end{aligned}$$

whereas the second derivative is given by

$$\begin{aligned} B^{2} = f_{\tau \tau }(r, u) = -\sum _{\nu = 1}^{m}\left( {\begin{array}{c}\beta \\ \nu \end{array}}\right) {{\,\textrm{Li}\,}}_{\beta -\nu +2}(-u)\Gamma (\beta -\nu + 3) r^{-\beta +\nu -3} + \mathcal {O}\bigl (r^{-3}\bigr ). \end{aligned}$$

For the third derivative occurring in the error term in (4.1), we have

$$\begin{aligned} \frac{\partial ^3 f(r+it, u)}{\partial t^3}&=-i\sum _{k\ge 1}\frac{k^{3}g(k)u^{-1}e^{k(r+it)}(1-u^{-1}e^{k(r+it)})}{(u^{-1}e^{k(r+it)}+1)^{3}}. \end{aligned}$$

We estimate this expression following [20]: Let $k_{0} = r^{-(1+c)}$ for some constant $c > 0$ and write $v = u^{-1}$ for short. We split the sum into two parts, according to whether $k \le k_{0}$ or not. For the sum over large *k*, we obtain

4.6 $$\begin{aligned}{} & {} {\left|-i\sum _{k> k_{0}}\frac{k^{3}g(k)ve^{k(r+it)}(1-ve^{k(r+it)})}{(ve^{k(r+it)}+1)^{3}} \right|} \le \sum _{k> k_{0}} \frac{k^{3}g(k)ve^{kr}(1+ve^{kr})}{{\left|ve^{k(r + it)} + 1 \right|}^{3}}\nonumber \\{} & {} \quad \le \sum _{k> k_{0}} \frac{k^{3}g(k)ve^{kr}(1 + ve^{kr})}{(ve^{kr} - 1)^{3}} =\mathcal {O}\left( \sum _{k > k_{0}} \frac{k^{3}g(k)}{e^{kr}}\right) =\mathcal {O}\left( r^{-\beta -3}\right) . \end{aligned}$$

For the remaining sum we note that

$$\begin{aligned} {\left|1 + ve^{k(r + it)} \right|} \ge (1 + ve^{kr})\cos \Bigl (\frac{kt}{2}\Bigr ). \end{aligned}$$

Therefore, we get

$$\begin{aligned}&{\left|-i\sum _{k\le k_{0}}\frac{k^{3}g(k)u^{-1}e^{k(r+it)}(1-u^{-1}e^{k(r+it)})}{(u^{-1}e^{k(r+it)}+1)^{3}} \right|}\le \\&\hspace{3cm}\le \sum _{k\le k_{0}}\frac{k^{3}g(k)ve^{kr}(1 + e^{kr})}{{\left|ve^{k(r+it)} + 1 \right|}^{3}}\\&\hspace{3cm}\le \sum _{k\le k_{0}} \frac{k^{3}g(k)ve^{kr}(1+e^{kr})}{(ve^{kr} + 1)^{3}}\Bigl (1+ O\bigl ((kt)^{2}\bigr )\Bigr )\\&\hspace{3cm}\le \sum _{k\le k_{0}} \frac{k^{3}g(k)}{ve^{kr}}\Bigl (1+ O\bigl ((kt)^{2}\bigr )\Bigr )\\&\hspace{3cm}\le \sum _{k\ge 1}\frac{k^{3}g(k)}{ve^{kr}} + O\Biggl (\sum _{k\ge 1}\frac{k^{5}g(k)t^{2}}{e^{kr}}\Biggr ). \end{aligned}$$

Using the Mellin transform and converse mapping, this results in

$$\begin{aligned} {\left|-i\sum _{k\le k_{0}}\frac{k^{3}g(k)u^{-1}e^{k(r+it)}(1-u^{-1}e^{k(r+it)})}{(u^{-1}e^{k(r+it)}+1)^{3}} \right|} =\mathcal {O}\left( r^{-\beta -3}+r^{-\beta -5}t^2\right) . \end{aligned}$$

By combining this with (4.6), we obtain

$$\begin{aligned}\frac{\partial ^3 f(r+it, u)}{\partial t^3}=\mathcal {O}\left( r^{-\beta -3}\right) \end{aligned}$$

for $$\left| t\right| \le r^{1+3\beta /7}$$.

All in all, this leads to the expansion

$$\begin{aligned} f(r + it, u) = f(r, u) - int - \frac{B^{2}}{2}t^{2} + \mathcal {O}\bigl (r^{-\beta -3}t^3\bigr ). \end{aligned}$$

For the integral $I_1$, we thus obtain

4.7 $$\begin{aligned}&\frac{e^{nr}}{2\pi }\int _{-t_{n}}^{t_{n}} \exp (int+f(r+it, u))\textrm{d}{t}\nonumber \\&\quad =\frac{e^{nr+f(r, u)}}{2\pi }\int _{-t_{n}}^{t_{n}} \exp \left( -\frac{B^2}{2}t^2+\mathcal {O}\bigl (r^{2\beta /7}\bigr )\right) \textrm{d}{t}\nonumber \\&\quad =\frac{e^{nr+f(r, u)}}{2\pi }\left( \int _{-t_{n}}^{t_{n}} \exp \left( -\frac{B^2}{2}t^2\right) \textrm{d}{t}\right) \Bigl (1+\mathcal {O}\bigl (r^{2\beta /7}\bigr )\Bigr ). \end{aligned}$$

Finally, we change the integral to a Gaussian integral and get

4.8 $$\begin{aligned} \int _{-t_{n}}^{t_{n}} \exp \left( -\frac{B^2}{2}t^2\right) \textrm{d}{t}&=\int _{-\infty }^{\infty } \exp \left( -\frac{B^2}{2}t^2\right) \textrm{d}{t} -2\int _{t_{n}}^{\infty } \exp \left( -\frac{B^2}{2}t^2\right) \textrm{d}{t}\nonumber \\&=\frac{\sqrt{2\pi }}{B}+\mathcal {O}\biggl ( \int _{t_{n}}^{\infty } \exp \biggl (-\frac{B^2r^{1+3\beta /7}}{2}t\biggr )\textrm{d}{t}\biggr )\nonumber \\&=\frac{\sqrt{2\pi }}{B}+\mathcal {O}\biggl ( r^{-1-3\beta /7}B^{-2}\exp \biggl (-\frac{r^{-\beta /7}}{2}\biggr )\biggr ). \end{aligned}$$

$$\square $$

### Estimate of $I_{2}$

Next, we prove the following asymptotic upper bound for the integral $I_{2}$.

#### Lemma 5

For $I_{2}$, it holds that

$$\begin{aligned} {\left|I_{2} \right|} =\mathcal {O}\left( \exp \bigl ( nr+f(r,u) - c_3r^{-\beta /7}\bigr )\right) , \end{aligned}$$

where $c_{3}$ is a constant uniformly in *u*.

For the proof of this estimate, we need the following two lemmas. The first lemma provides an upper bound for some exponential that will occur later on, whereas the second one says that ${\left|Q(e^{-r-it},u) \right|}$ is small compared to $Q(e^{r},u)$. These results are the main ingredients for the proof of Lemma 5.

#### Lemma 6

(Li–Chen [17, Lemma 2.5]) Let $\beta \ge 1$. For $\sigma >0$, we have

$$\begin{aligned} \sum _{k\ge 1}k^{\beta -1}e^{-k\sigma }(1-\cos (ky)) \ge \rho \biggl (\frac{e^{-\sigma }}{(1-e^{-\sigma })^\beta }- \frac{e^{-\sigma }}{{\left|1-e^{-\sigma -iy} \right|}^\beta }\biggr ), \end{aligned}$$

where $\rho$ is a positive constant depending only on $\beta$.

#### Lemma 7

For any real *y* with $t_{0}\le {\left|y \right|} \le \pi$, we derive

$$\begin{aligned}\frac{{\left|Q(e^{-(r+iy)},u) \right|}}{Q(e^{-r},u)} \le \exp \biggl (-\frac{2u(\beta -1)}{(1+u)^{2}}\rho \Bigl (\frac{1}{4} r^{-\beta /7}\Bigr )\biggr )\end{aligned}$$

for some constant $\rho$ depending only on $\beta$.

#### Proof

First of all, we note thatBy the mean value theorem, there exists $\xi \in (k,k+1)$ such that

$$\begin{aligned} (k+1)^{\beta } - k^{\beta } = \beta \xi ^{\beta -1}, \end{aligned}$$

which leads to

4.9 $$\begin{aligned} g(k) \ge \beta \xi ^{\beta -1} - 1 = (\beta -1)\xi ^{\beta -1} + \xi ^{\beta -1} - 1 > (\beta -1)k^{\beta -1}; \end{aligned}$$

see also Li and Chen [17, Proof of Lemma 2.6]. Moreover, we have

$$\begin{aligned} {\left|1+ue^{-k(r+iy)} \right|}^{2}&=(1+ue^{-kr-kiy})(1+ue^{-kr+kiy})\\&=1+ue^{-kr-kiy}+ue^{-kr+kiy}+u^2e^{-2kr}\\&=1+2ue^{-kr}+(ue^{-kr})^2-2ue^{-kr}+2ue^{-kr}\cos (ky)\\&=(1+ue^{-kr})^2-2ue^{-kr}(1-\cos (ky)). \end{aligned}$$

Using this, it holds that

$$\begin{aligned} \biggl (\frac{{\left|Q(e^{-(r+iy)},u) \right|}}{Q(e^{-r},u)}\biggr )^2&=\prod _{k\ge 1}\biggl (1-\frac{2ue^{-kr}(1-\cos (ky))}{(1+ue^{-kr})^2}\biggr )^{g(k)}\\&=\exp \biggl (\sum _{k\ge 1}g(k)\log \biggl (1-\frac{2ue^{-kr}(1-\cos (ky))}{(1+ue^{-kr})^2}\biggr )\biggr )\\&\le \exp \biggl (-\sum _{k\ge 1}g(k)\frac{2ue^{-kr}(1-\cos (ky))}{(1+ue^{-kr})^2}\biggr )\\&\le \exp \biggl (-\frac{2u}{(1+u)^2}\sum _{k\ge 1}g(k)e^{-kr}(1-\cos (ky))\biggr )\\&\le \exp \biggl (-\frac{2u(\beta -1)}{(1+u)^2}\sum _{k\ge 1}k^{\beta -1}e^{-kr}(1-\cos (ky))\biggr )\\&\le \exp \biggl (-\frac{2u(\beta -1)}{(1+u)^{2}}\rho \biggl (\frac{e^{-r}}{(1-e^{-r})^{\beta }} - \frac{e^{-r}}{{\left|1-e^{-r-iy} \right|}^{\beta }}\biggr )\biggr ), \end{aligned}$$

where the last estimate follows by Lemma 6.

Following the lines of Li and Chen [17] again, we further have

$$\begin{aligned} {\left|1 - e^{-r - it} \right|}^{\beta }&= (1 - 2e^{-r}\cos t + e^{-2r})^{\beta /2}\\&= ((1 - e^{-r})^{2} +2e^{-r}(1 - \cos t))^{\beta /2}\\&\ge (1-e^{-r})^{\beta }\Bigl (1 + \frac{2e^{-r}}{(1 - e^{-r})^{2}}(1 - \cos t_{n})\Bigr )^{\beta /2} \end{aligned}$$

for $$t_{n}\le {\left|t \right|}\le \pi $$. Since

$$\begin{aligned} 1 - \cos t_{n} = \frac{1}{2} t_{n}^{2} + \mathcal {O}(t_{n}^{4}) = \frac{1}{2} r^{2+6\beta /7}+ \mathcal {O}(r^{4 + 12\beta /7}) \end{aligned}$$

and $e^{-r} = 1 - r + \mathcal {O}(r^{2})$, we find

$$\begin{aligned} \frac{2e^{r}}{(1-e^{r})^{2}}(1 - \cos t_{n})&= \frac{2(1 + \mathcal {O}(r))(\frac{1}{2} r^{2 + 6\beta /7} + \mathcal {O}(r^{4+12\beta /7}))}{r^{2}(1 + \mathcal {O}(r))}\\ {}&= r^{6\beta /7}(1 + \mathcal {O}(r)). \end{aligned}$$

This further implies

$$\begin{aligned} {\left|1 - e^{-r - it} \right|}^{\beta }&\ge (1 - e^{-r})^{\beta }\bigl (1 + r^{6\beta /7}(1 + \mathcal {O}(r))\bigr )^{\beta /2}\\&= (1 - e^{-r})^{\beta }\Bigl (1 + \frac{\beta }{2}r^{6\beta /7} + \mathcal {O}(r^{6\beta /7 + 1} + r^{12\beta /7})\Bigr ). \end{aligned}$$

This lower bound results in

$$\begin{aligned}&\frac{e^{r}}{(1-e^{-r})^\beta }-\frac{e^{r}}{\left| 1-e^{-r-it}\right| ^\beta }\\&\quad \ge \frac{e^{r}}{(1-e^{-r})^\beta }\left( 1-\frac{1}{1+\frac{\beta }{2}r^{6\beta /7}+\mathcal {O}\left( r^{6\beta /7+1} + r^{12\beta /7}\right) }\right) \\&\quad =\frac{e^{r}}{(1-e^{-r})^\beta }\left( \frac{\beta }{2}r^{6\beta /7}+\mathcal {O}\left( r^{6\beta /7+1} + r^{12\beta /7}\right) \right) \\&\quad =\frac{1+\mathcal {O}(r)}{r^\beta (1-\mathcal {O}(r))}\left( \frac{\beta }{2}r^{6\beta /7}+\mathcal {O}\left( r^{6\beta /7+1} + r^{12\beta /7}\right) \right) \\&\quad =\frac{\beta }{2} r^{6\beta /7-\beta }+\mathcal {O}\bigl (r^{6\beta /7+1-\beta } + r^{12\beta /7}\bigr ) \end{aligned}$$

and finally

$$\begin{aligned} \frac{e^{r}}{(1-e^{-r})^\beta }-\frac{e^{r}}{\left| 1-e^{-r-it}\right| ^\beta } \ge \frac{1}{4} r^{6\beta /7-\beta } \end{aligned}$$

for sufficiently large *n*. So we consequently obtain

$$\begin{aligned} \biggl (\frac{{\left|Q(e^{-(r+iy)}, u) \right|}}{Q(e^{-r}, u)}\biggr )^2 \le \exp \biggl (-\frac{2u(\beta -1)}{(1+u)^{2}}\rho \Bigl (\frac{1}{4} r^{6\beta /7-\beta }\Bigr )\biggr ), \end{aligned}$$

completing the proof. $\square$

#### Proof of Lemma 5

By the definition of $I_{2}$, it holds that

$$\begin{aligned} {\left|I_2 \right|} \le \frac{e^{nr}}{\pi }\int _{t_n}^\pi {\left|Q(e^{-r-it}, u) \right|}\textrm{d}{t} = \frac{e^{nr+f(r, u)}}{\pi }\int _{t_n}^\pi \frac{{\left|Q(e^{-r-it}, u) \right|}}{Q(e^{-r},u)}\textrm{d}{t}. \end{aligned}$$

An application of Lemma 7 thus yields

4.10 $$\begin{aligned} {\left|I_2 \right|} =\mathcal {O}\left( \exp \left( nr+f(r,u) - c_3r^{-\beta /7}\right) \right) \end{aligned}$$

for a certain constant $c_3$ uniformly in *u*, as stated. $\square$

### Estimate of $Q_n(u)$ and Moment-Generating Function

After estimating the main term and the contribution away from the real axis, we put (4.8) and (4.10) together and get

$$\begin{aligned} Q_n(u) =\frac{e^{nr+f(u,r)}}{\sqrt{2\pi B}}\bigl (1+\mathcal {O}(r^{2\beta /7})\bigr ). \end{aligned}$$

The plan for the last part of the proof is to consider the moment-generating function using this asymptotic expansion. This will prove the central limit theorem. Finally, at the end of this section, we use the Chernoff bound in order to obtain the desired tail estimates.

Now we consider the moment-generating function for the random variable $\varpi _n$ (the number of summands in a random partition of *n*). To this end, let $M_n(t)={\mathbb {E}}(e^{(\varpi _n-\mu _n)t/\sigma _n})$, where *t* is real and $\mu _n$ and $\sigma _n$ are the mean and the standard deviation as defined in (2.1) and (2.2), respectively. Then the following estimate holds.

#### Lemma 8

For bounded *t*, it holds that

$$\begin{aligned} M_n(t) = \exp \left( \frac{t^{2}}{2} + \mathcal {O}(n^{-2\beta /(7\beta +7)})\right) \end{aligned}$$

as $$n\rightarrow \infty $$.

#### Proof

First of all, we observe that

4.11 $$\begin{aligned} M_n(t)&=\exp \left( -\frac{\mu _nt}{\sigma _n}\right) \frac{Q_n(e^{t/\sigma _n})}{Q_n(1)}\nonumber \\&=\sqrt{\frac{B^2(r(1,n),1)}{B^2(r(n, e^{t/\sigma _n}), e^{t/\sigma _n})}} \exp \Bigl (-\frac{\mu _nt}{\sigma _n}+nr(n,e^{t/\sigma _n})\nonumber \\&\qquad +f(r(n, e^{t/\sigma _n}),e^{t/\sigma _n})-nr(n,1)-f(r(n,1),1)+\mathcal {O}(r^{2\beta /7})\Bigr ).\qquad \end{aligned}$$

Instead of representing the function with respect to *u*, we interpret $r=r(n,u)$ as a function of *n* and *u* and use implicit differentiation on (4.5) as in Madritsch and Wagner [20], and obtain

$$\begin{aligned} r_u=r_u(n,u)=-\frac{f_{u\tau }(r,u)}{f_{\tau \tau }(r,u)}= \frac{\sum _{k\ge 1}\frac{kg(k)e^{kr}}{(e^{kr}+u)^2}}{\sum _{k\ge 1}\frac{uk^2g(k)e^{kr}}{(e^{kr}+u)^2}} \end{aligned}$$

and similarly

$$\begin{aligned} r_{uu}&= r_{uu}(n,u)\\ {}&= \frac{-f_{\tau \tau \tau }(r,u)f_{u\tau }(r,u)^2+2f_{u\tau \tau }(r,u)f_{u\tau }(r,u)f_{\tau \tau }(r,u)-f_{uu\tau }(r,u)f_{\tau \tau }(r,u)^2}{f_{\tau \tau }(r,u)^3} \end{aligned}$$

as well as

$$\begin{aligned} r_{uuu}&= r_{uuu}(n,u)\\ {}&= f_{\tau \tau }(r,u)^{-5} \Big (-f_{uuu\tau }(r,u) f_{\tau \tau }(r,u)^4 \\&\qquad + \left( 3 f_{uu\tau \tau }(r,u) f_{u\tau }(r,u)+3 f_{uu\tau }(r,u) f_{u\tau \tau }(r,u)\right) f_{\tau \tau }(r,u)^3 \\&\qquad +\left( -3f_{u\tau \tau \tau }(r,u) f_{u\tau }(r,u)^2-6 f_{u\tau \tau }(r,u)^2 f_{u\tau }(r,u)\right. \\&\qquad \qquad \left. -3f_{uu\tau }(r,u) f_{\tau \tau \tau }(r,u) f_{u\tau }(r,u)\right) f_{\tau \tau }(r,u)^2 \\&\qquad +\left( f_{\tau \tau \tau \tau }(r,u) f_{u\tau }(r,u)^3+9 f_{u\tau \tau }(r,u) f_{\tau \tau \tau }(r,u) f_{u\tau }(r,u)^2\right) f_{\tau \tau }(r,u) \\&\qquad -3 f_{u\tau }(r,u)^3 f_{\tau \tau \tau }(r,u)^2 \Big ). \end{aligned}$$

We now need estimates for the partial derivatives of *f*. Estimates for partial derivatives with respect to $\tau$ follow from our considerations in Section 4.1. For derivatives with respect to *u*, we take the derivative of the corresponding Mellin transform and then obtain the estimate via converse mapping again. Let us exemplarily illustrate this approach for $f_{u\tau }$: By (4.4), $f_{\tau }$ is given by

$$\begin{aligned} f_{\tau }(\tau , u) = \sum _{\nu =1}^{m}\left( {\begin{array}{c}\beta \\ \nu \end{array}}\right) h_{\beta -\nu , 1}(\tau , u) + \mathcal {O}(h_{0, 1}(\tau , u)). \end{aligned}$$

The Mellin transform of $h_{\beta - \nu , 1}$ is given by

$$\begin{aligned} H_{\beta - \nu , 1}(s, u) = \zeta (s - \beta + \nu - 1){{\,\textrm{Li}\,}}_{s}(-u)\Gamma (s). \end{aligned}$$

Taking the derivative of $H_{\beta - \nu , 1}$ with respect to *u* thus yields

$$\begin{aligned} \frac{\partial H_{\beta - \nu , 1}}{\partial u}\,\!(s, u) = \frac{1}{u}\zeta (s - \beta + \nu - 1){{\,\textrm{Li}\,}}_{s-1}(-u)\Gamma (s). \end{aligned}$$

Consequently, converse mapping implies for $f_{\tau u}$ that

$$\begin{aligned} \begin{aligned} f_{\tau u}(r, u)&= \frac{1}{u}\sum _{\nu =1}^{m}\left( {\begin{array}{c}\beta \\ \nu \end{array}}\right) {{\,\textrm{Li}\,}}_{\beta - \nu + 1}(-u)\Gamma (\beta - \nu + 2)r^{-\beta + \nu - 2} + \mathcal {O}(r^{-3})\\&\sim \frac{\beta }{u} {{\,\textrm{Li}\,}}_{\beta }(-u)\Gamma (\beta +1)r^{-(\beta +1)}=\mathcal {O}\left( n\right) . \end{aligned} \end{aligned}$$

In a similar manner, we determine estimates for the other partial derivatives and obtain

$$\begin{aligned} f_{u}(r, u)&= \sum _{k\ge 1}\frac{g(k)}{e^{kr} + u} \sim -\frac{1}{u}{{\,\textrm{Li}\,}}_{\beta }(-u)\Gamma (\beta +1)r^{-\beta } =\mathcal {O}\left( n^{\beta /(\beta + 1)}\right) ,\\ f_{uu}(r, u)&= -\sum _{k\ge 1}\frac{g(k)}{(e^{kr} + u)^{2}} \sim \frac{1}{u^2}\left( {{\,\textrm{Li}\,}}_{\beta }(-u)+{{\,\textrm{Li}\,}}_{\beta -1}(-u)\right) \Gamma (\beta +1)r^{-\beta } \\&=\mathcal {O}\left( n^{\beta /(\beta + 1)}\right) ,\\ f_{\tau \tau }(r,u)&= \sum _{k\ge 1} \frac{uk^2g(k)e^{kr}}{(e^{kr}+u)^2} \sim -\beta (\beta +1){{\,\textrm{Li}\,}}_{\beta +1}(-u)\Gamma (\beta +1)r^{-(\beta +2)} \\&=\mathcal {O}\left( n^{(\beta +2)/(\beta +1)}\right) , \\ f_{uuu}(r, u)&= \sum _{k\ge 1}\frac{2g(k)}{(e^{kr} + u)^{3}} =\mathcal {O}\left( r^{-\beta }\right) =\mathcal {O}\left( n^{\beta /(\beta + 1)}\right) ,\\ f_{uu\tau }(r,u)&= \sum _{k\ge 1} \frac{2kg(k)e^{kr}}{(e^{kr}+u)^3} =\mathcal {O}\left( r^{-(\beta +1)}\right) =\mathcal {O}\left( n\right) ,\\ f_{u\tau \tau }(r,u)&= \sum _{k\ge 1} \frac{k^2g(k)e^{kr}(e^{kr}-u)}{(e^{kr}+u)^3} =\mathcal {O}\left( r^{-(\beta +2)}\right) =\mathcal {O}\left( n^{(\beta +2)/(\beta +1)}\right) , \\ f_{\tau \tau \tau }(r,u)&= - \sum _{k\ge 1} \frac{uk^3g(k)e^{kr}(e^{kr}-u)}{(e^{kr}+u)^3} =\mathcal {O}\left( r^{-(\beta +3)}\right) =\mathcal {O}\left( n^{(\beta +3)/(\beta +1)}\right) , \\ f_{uuu\tau }(r,u)&= - \sum _{k\ge 1} \frac{6kg(k)e^{kr}}{(e^{kr}+u)^4} =\mathcal {O}\left( r^{-(\beta +1)}\right) =\mathcal {O}\left( n\right) , \\ f_{uu\tau \tau }(r,u)&= - \sum _{k\ge 1} \frac{2k^2g(k)e^{kr}(2e^{kr}-u)}{(e^{kr}+u)^4} =\mathcal {O}\left( r^{-(\beta +2)}\right) =\mathcal {O}\left( n^{(\beta +2)/(\beta +1)}\right) , \\ f_{u\tau \tau \tau }(r,u)&= - \sum _{k\ge 1} \frac{k^3g(k)e^{kr}(e^{2kr}-4ue^{kr}+u^2)}{(e^{kr}+u)^4} \\&=\mathcal {O}\left( r^{-(\beta +3)}\right) =\mathcal {O}\left( n^{(\beta +3)/(\beta +1)}\right) , \\ f_{\tau \tau \tau \tau }(r,u)&= \sum _{k\ge 1} \frac{uk^4g(k)e^{kr}(e^{2kr}-4ue^{kr}+u^2)}{(e^{kr}+u)^4}\\&=\mathcal {O}\left( r^{-(\beta +4)}\right) =\mathcal {O}\left( n^{(\beta +4)/(\beta +1)}\right) . \end{aligned}$$

From these estimates it follows that $r_u,r_{uu},r_{uuu}=\mathcal {O}\left( n^{-1/(\beta + 1)}\right)$ uniformly in *u*. Expanding $r(n,e^{t/\sigma _n})$ and $f(r(e^{t/\sigma _n},n),e^{t/\sigma _n})$ around $t=0$ yields

$$\begin{aligned} r(n,e^{t/\sigma _n}) - r(n,1)= & {} r_u(n,1)\cdot \frac{t}{\sigma _n} +\frac{r_u(n,1)+r_{uu}(n,1)}{2}\cdot \left( \frac{t}{\sigma _n}\right) ^2 \\{} & {} +\mathcal {O}\left( n^{-1/(\beta +1)}\frac{{\left|t \right|}^3}{\sigma _n^3}\right) \end{aligned}$$

and

$$\begin{aligned}&f(r(e^{t/\sigma _n},n),e^{t/\sigma _n}) - f(r(n,1),1)\\&\quad =\left( f_{\tau }(r(n,1),1)r_u(n,1)+f_u(r(n,1),1)\right) \cdot \frac{t}{\sigma _n}\\&\quad \ \quad +\frac{1}{2}\left( f_{\tau }(r(n,1),1)(r_u(n,1) + r_{uu}(n,1)) +f_{\tau \tau }(r(n,1),1)r_u(n,1)^2\right. \\&\quad \ \quad \left. + 2f_{u\tau }(r(n,1),1)r_u(n,1) + f_u(r(n,1),1)+f_{uu}(r(n,1),1)\right) \cdot \left( \frac{t}{\sigma _n}\right) ^2\\&\quad \quad \ +\mathcal {O}\left( n^{\beta /(\beta +1)}\frac{{\left|t \right|}^3}{\sigma _n^3}\right) , \end{aligned}$$

respectively.

By plugging these expansions into the exponential of (4.11), we get

$$\begin{aligned}{} & {} \bigl (nr_u(n,1)+f_\tau (\eta ,1)r_u(n,1)+f_u(\eta ,1)-\mu _n\bigr ) \cdot \frac{t}{\sigma _n}\\{} & {} \quad +\frac{1}{2}\bigl (n(r_u(n,1)\!+\!r_{uu}(n,1)\bigr )\!+\! f_{\tau }(\eta ,1)\bigl (r_u(n,1)\!+\!r_{uu}(n,1)\bigr ) \!+\!f_{\tau \tau }(\eta ,1)r_u(n,1)^2\\{} & {} \quad + 2f_{u\tau }(\eta ,1)r_{u}(n,1) + f_{u}(\eta , 1) + f_{uu}(\eta , 1)\bigr ) \cdot \left( \frac{t}{\sigma _n}\right) ^2\\{} & {} \quad +\mathcal {O}\left( n^{\beta /(\beta + 1)}\frac{{\left|t \right|}^3}{\sigma _n^3}+n^{-2\beta /(7\beta +7)}\right) , \end{aligned}$$

where we have written $\eta =r(n,1)$ for short. Recalling that $n=-f_\tau (\eta ,1)$ and that $r_u(n,1)=-\frac{f_{u\tau }(\eta ,1)}{f_{\tau \tau }(\eta ,1)}$, we can simplify the last expression in order to obtain

$$\begin{aligned}{} & {} \left( f_u(\eta ,1)-\mu _n\right) \cdot \frac{t}{\sigma _n} +\frac{1}{2}\left( f_u(\eta ,1)+f_{uu}(\eta ,1) -\frac{f_{u\tau }(\eta ,1)^2}{f_{\tau \tau }(\eta ,1)}\right) \cdot \left( \frac{t}{\sigma _n}\right) ^2\\{} & {} \quad +\mathcal {O}\biggl (n^{\beta /(\beta + 1)}\frac{{\left|t \right|}^3}{\sigma _n^3}+n^{-2\beta /(7\beta +7)}\biggr ). \end{aligned}$$

In a similar way we get that

$$\begin{aligned}\frac{B^2(r(n,1),1)}{B^2(r(n,e^{t/\sigma _n}), e^{t/\sigma _n})} =1+\mathcal {O}\left( \frac{{\left|t \right|}}{\sigma _n}\right) .\end{aligned}$$

Thus, we obtain the following asymptotic formula for the moment-generating function in (4.11):

$$\begin{aligned}{} & {} M_n(t)=\exp \biggl (\bigl (f_u(\eta ,1)-\mu _n\bigr )\cdot \frac{t}{\sigma _n} +\frac{1}{2}\left( f_u(\eta ,1)+f_{uu}(\eta ,1) -\frac{f_{u\tau }(\eta ,1)^2}{f_{\tau \tau }(\eta ,1)}\right) \cdot \left( \frac{t}{\sigma _n}\right) ^2\\{} & {} \quad +\mathcal {O}\biggl (\frac{{\left|t \right|}}{\sigma _n}+n^{\frac{\beta }{\beta + 1}}\frac{{\left|t \right|}^3}{\sigma _n^3}+n^{-2\beta /(7\beta +7)}\biggr )\biggr ). \end{aligned}$$

Recall that we chose $\mu _{n}$ and $\sigma _{n}$ in (2.1) and (2.2) such that

$$\begin{aligned} \mu _n&=f_{u}(\eta ,1)=\sum _{k\ge 1}\frac{g(k)}{e^{\eta k}+1}\quad \text {and}\\ \sigma _n^2&=f_{u}(\eta ,1)+f_{uu}(\eta ,1)-\frac{f_{u\tau }(\eta ,1)^2}{f_{\tau \tau }(\eta ,1)} =\sum _{k\ge 1}\frac{g(k)e^{\eta k}}{(e^{\eta k}+1)^2} -\frac{\left( \sum _{k\ge 1}\frac{g(k)ke^{\eta k}}{(e^{\eta k}+1)^2}\right) ^2}{\sum _{k\ge 1}\frac{g(k)k^2e^{\eta k}}{(e^{\eta k}+1)^2}}. \end{aligned}$$

By definition of $\mu _n$ and $\sigma _n^2$ in (2.1) and (2.2), respectively, we deduce that

$$\begin{aligned} M_n(t)&=\exp \left( \frac{t^2}{2}+\mathcal {O}\bigl (n^{-\beta /(2\beta + 2)}{\left|t \right|} + n^{-\beta /(2\beta + 2)}{\left|t \right|}^{3} + n^{-2\beta /(7\beta +7)}\bigr )\right) \\&= \exp \left( \frac{t^2}{2}+\mathcal {O}\bigl (n^{-\beta /(2\beta + 2)}({\left|t \right|} + {\left|t \right|}^{3}) + n^{-2\beta /(7\beta +7)}\bigr )\right) \\&= \exp \left( \frac{t^{2}}{2} + \mathcal {O}(n^{-2\beta /(7\beta +7)})\right) \end{aligned}$$

for bounded *t*. $$\square $$

By the previous lemma and Curtiss’ theorem [5], it follows that the distribution of $\varpi _{n}$ is indeed asymptotically normal. For the remaining parts, we first show that the two asymptotic formulas in (2.1) and (2.2) hold for $\mu _n$ and $\sigma _n$, respectively. In particular, we show the existence of two positive constants $c_1$ and $c_2$ such that

$$\begin{aligned} \mu _n \sim c_1n^{\frac{\beta }{\beta +1}}\quad \text {and}\quad \sigma _n^2 \sim c_2\eta ^{\frac{\beta }{\beta +1}}. \end{aligned}$$

Our Mellin transform techniques from above show that

$$\begin{aligned} \mu _n&\sim -{{\,\textrm{Li}\,}}_{\beta }(-1)\Gamma (\beta +1)\eta ^{-\beta }\\ \end{aligned}$$

and

$$\begin{aligned} \sigma _n^2&\sim \left( -{{\,\textrm{Li}\,}}_{\beta -1}(-1)+\frac{\beta }{\beta +1}\frac{{{\,\textrm{Li}\,}}_{\beta }(-1)^2}{{{\,\textrm{Li}\,}}_{\beta +1}(-1)} \right) \Gamma (\beta +1)\eta ^{-\beta }. \end{aligned}$$

We may use the identity

$$\begin{aligned} -{{\,\textrm{Li}\,}}_s(-1) =\left( 1-2^{1-s}\right) \zeta (s) \end{aligned}$$

to relate these formulas to the Riemann zeta function. Thus we get

4.12 $$\begin{aligned} \mu _n&\sim \beta \left( 1-2^{1-\beta }\right) \zeta (\beta )\Gamma (\beta )\eta ^{-\beta } \end{aligned}$$

and

4.13 $$\begin{aligned} \sigma _n^2&\sim \left( \left( 1-2^{2-\beta }\right) \zeta (\beta -1) -\frac{\beta }{\beta +1}\frac{(1-2^{1-\beta })^2\zeta (\beta )^2}{(1-2^{-\beta })\zeta (\beta +1)}\right) \Gamma (\beta +1)\eta ^{-\beta }. \end{aligned}$$

From (4.5) we get by Lagrange inversion that

$$\begin{aligned} \eta ^{-1}=r(n,1)^{-1}&\sim \left( -\beta {{\,\textrm{Li}\,}}_{\beta +1}(-1)\Gamma (\beta +1)\right) ^{-\frac{1}{\beta +1}}n^{\frac{1}{\beta +1}}\\&=\left( \left( 1-2^{-\beta }\right) \zeta (\beta +1)\Gamma (\beta +1)\right) ^{-\frac{1}{\beta +1}}n^{\frac{1}{\beta +1}} \end{aligned}$$

and substituting this in (4.12) and (4.13), respectively, yields

$$\begin{aligned}\mu _n\sim c_1 n^{\frac{\beta }{\beta +1}} \quad \text {and}\quad \sigma _n^2\sim c_2 n^{\frac{\beta }{\beta +1}}.\end{aligned}$$

We still need to show that $c_1$ and $c_2$ are both positive. For $c_1$ we note that every term $g(k)/(e^{\eta k}+1)$ is positive and therefore the whole sum is positive. For $\sigma _n^2$ it is not so obvious that $c_2$ does not vanish identically. Therefore we consider the numerator and denominator of $\sigma _n^2$ separately. For the numerator we get

$$\begin{aligned}&\left( \sum _{k\ge 1}g(k)q(\eta k)\right) \left( \sum _{k\ge 1}k^2g(k)q(\eta k)\right) -\left( \sum _{k\ge 1}kg(k)q(\eta k)\right) ^2\\&\quad =\sum _{k_1\ge 1}\sum _{k_2\ge 1} (k_2^2-k_1k_2)g(k_1)g(k_2)q(\eta k_1)q(\eta k_2)\\&\quad =\sum _{k_1\ge 1}\sum _{k_2>k_1} (k_2-k_1)^2g(k_1)g(k_2)q(\eta k_1)q(\eta k_2)\\&\quad =\frac{1}{2}\sum _{k_1\ge 1}\sum _{k_2\ge 1} (k_2-k_1)^2g(k_1)g(k_2)q(\eta k_1)q(\eta k_2), \end{aligned}$$

where we have written $q(x)=\frac{e^x}{(e^x+1)^2}$ for short. Let $C>1$ be an arbitrary constant. Then we can estimate this by

$$\begin{aligned}&\frac{1}{2}\sum _{k_1\ge 1}\sum _{k_2\ge 1} (k_2-k_1)^2g(k_1)g(k_2)q(\eta k_1)q(\eta k_2)\\&\quad \ge \frac{1}{2}\sum _{k_1\le \frac{\eta ^{-1}}{2}}\sum _{\eta ^{-1}\le k_2\le C\eta ^{-1}} (k_2-k_1)^2g(k_1)g(k_2)q(\eta k_1)q(\eta k_2)\\&\quad \ge \frac{1}{2}\sum _{k_1\le \frac{\eta ^{-1}}{2}}\sum _{\eta ^{-1}\le k_2\le C\eta ^{-1}} \frac{1}{4\eta ^2} g(k_1)g(k_2)q(\eta k_1)q(\eta k_2)\\&\quad \gg \eta ^{-2}\left( \sum _{k\le \frac{\eta ^{-1}}{2}} g(k)\right) \left( \sum _{\eta ^{-1}\le k\le C\eta ^{-1}} g(k)\right) \\&\quad \gg \eta ^{-2-2\beta }, \end{aligned}$$

where we have used that $g(k)\gg k^{\beta -1}$ by (4.9). For the denominator we already have shown that it is $\ll \eta ^{-(\beta +2)}$. Thus we have $\sigma _n^2\gg \eta ^{-\beta }$ and $c_2>0$.

For the asymptotic equivalences

$$\begin{aligned} {\mathbb {E}}(\varpi _n)\sim \mu _n\quad \text {and}\quad {\mathbb {V}}(\varpi _n)\sim \sigma _n^2\end{aligned}$$

we apply Hwang’s method used in the proof of Theorem 1 in [14].

Finally we turn our attention to the tails. We again follow Hwang [14] and obtain for $t = o(n^{\alpha /(6\alpha +6)})$ that

$$\begin{aligned} {\mathbb {P}}\left( \frac{\varpi _{n} - \mu _n}{\sigma _n}\ge x\right)&\le e^{-tx}M_n(t)\\&= e^{-tx+t^2/2}\left( 1+\mathcal {O}\bigl (n^{-\beta /(2\beta + 2)}({\left|t \right|} + {\left|t \right|}^{3}) + n^{-2\beta /(7\beta +7)}\bigr )\right) \end{aligned}$$

by the Chernoff bound. For the original inequality of Chernoff we refer to [4] and we remark here that Herman Chernoff has celebrated his 100 anniversary on July 1, 2023.

Therefore, let $T=n^{\beta /(6\beta +6)}/\log n$. Then for $x\le T$ we set $t=x$ and obtain

$$\begin{aligned} {\mathbb {P}}\left( \frac{\varpi _{n} - \mu _n}{\sigma _n}\ge x\right) \le e^{-x^2/2}\left( 1+\mathcal {O}\left( (\log n)^{-3}\right) \right) . \end{aligned}$$

For $x\ge T$ we set $t=T$ yielding

$$\begin{aligned} {\mathbb {P}}\left( \frac{\varpi _{n} - \mu _n}{\sigma _n}\ge x\right) \le e^{-Tx/2}\left( 1+\mathcal {O}\left( (\log n)^{-3}\right) \right) . \end{aligned}$$

We can estimate the probability ${\mathbb {P}}\bigl (\frac{\varpi _{n} - \mu _n}{\sigma _n}\le -x\bigr )$ in a similar way.
